# Supplementary material for: Mechanisms governing the pioneering and redistribution capabilities of the non-classical pioneer PU.1
Source: Nat Commun. 2020 Jan 21;11:402. doi: 10.1038/s41467-019-13960-2 (PMC6972792; doi:10.1038/s41467-019-13960-2)
Supplement: Supplementary file 7 — Source data [file 41467_2019_13960_MOESM7_ESM.zip › Source_Data/FigureS2/FigureS2B_MotifScanOutput/KmeansCluster1/homerResults.html]

/misc/data/analysis/project\_PU1/CTV1/motifs/basic/PU1\_Kmeans14.1/final - Homer de novo Motif Results


# Homer *de novo* Motif Results (/misc/data/analysis/project\_PU1/CTV1/motifs/basic/PU1\_Kmeans14.1/final)

Known Motif Enrichment Results  
Gene Ontology Enrichment Results  
If Homer is having trouble matching a motif to a known motif, try copy/pasting the matrix file into
STAMP  
More information on motif finding results: HOMER
| Description of Results
| Tips
  
Total target sequences = 5091  
Total background sequences = 39647  
\* - possible false positive  

|  |  |  |  |  |  |  |  |  |
| --- | --- | --- | --- | --- | --- | --- | --- | --- |
| Rank | Motif | P-value | log P-pvalue | % of Targets | % of Background | STD(Bg STD) | Best Match/Details | Motif File |
| 1 | A T G C C G T A A T G C A C G T A C G T A G T C A G T C A G C T A G T C G C A T G C A T G C A T G A C T A G C T | 1e-3599 | -8.289e+03 | 83.50% | 4.21% | 19.4bp (73.2bp) | SpiB(ETS)/OCILY3-SPIB-ChIP-Seq(GSE56857)/Homer(0.966) More Information \| Similar Motifs Found | motif file (matrix) |
| 2 | C G A T A C G T A T G C A T G C G A T C A T G C G C A T C G A T | 1e-1192 | -2.747e+03 | 60.01% | 12.03% | 44.2bp (76.0bp) | SPIB/MA0081.1/Jaspar(0.822) More Information \| Similar Motifs Found | motif file (matrix) |
| 3 | A C G T A C G T A G T C A G T C A C G T T A C G A C G T A C G T A C G T | 1e-688 | -1.586e+03 | 27.48% | 2.90% | 39.0bp (75.1bp) | Ets1-distal(ETS)/CD4+-PolII-ChIP-Seq(Barski\_et\_al.)/Homer(0.817) More Information \| Similar Motifs Found | motif file (matrix) |
| 4 | C T G A T C G A T C A G C T A G T C A G C T A G T C G A T C G A T C A G | 1e-630 | -1.453e+03 | 48.67% | 14.18% | 49.4bp (88.7bp) | RO3G\_00049(RRM)/Rhizopus\_oryzae-RNCMPT00205-PBM/HughesRNA(0.805) More Information \| Similar Motifs Found | motif file (matrix) |
| 5 | C T A G C G T A C T G A A C T G C T G A T C A G A C T G C G T A | 1e-392 | -9.029e+02 | 40.05% | 13.87% | 48.5bp (91.0bp) | Unknown4/Arabidopsis-Promoters/Homer(0.765) More Information \| Similar Motifs Found | motif file (matrix) |
| 6 | A G T C C G A T A G C T T G A C G A C T A G C T T G A C A G C T A G C T G A T C A G C T A G C T | 1e-275 | -6.353e+02 | 23.53% | 6.50% | 53.9bp (98.0bp) | Unknown4/Arabidopsis-Promoters/Homer(0.706) More Information \| Similar Motifs Found | motif file (matrix) |
| 7 | G A C T A G T C G A T C G A C T A G T C G A T C G A C T A G T C G A T C G A C T A G T C G A T C G A C T A G T C | 1e-173 | -4.003e+02 | 60.60% | 39.75% | 46.7bp (96.6bp) | ZNF263/MA0528.1/Jaspar(0.769) More Information \| Similar Motifs Found | motif file (matrix) |
| 8 | A T G C G C A T G T A C C G A T C A G T G T A C G T A C G C A T A G T C G T A C G C A T A G T C C G A T A C G T | 1e-117 | -2.707e+02 | 9.37% | 2.29% | 54.9bp (80.4bp) | PF10\_0068(RRM)/Plasmodium\_falciparum-RNCMPT00199-PBM/HughesRNA(0.610) More Information \| Similar Motifs Found | motif file (matrix) |
| 9 | C T A G C T A G T C G A T C G A C T A G C T A G T C G A C T G A C T A G C T A G T C G A T C G A T C A G | 1e-102 | -2.371e+02 | 21.02% | 9.97% | 50.9bp (109.7bp) | EWSR1-FLI1/MA0149.1/Jaspar(0.802) More Information \| Similar Motifs Found | motif file (matrix) |
| 10 | A T G C G T C A A T G C A C G T A C G T A G T C A C G T T C A G A G T C G C A T C G A T G A C T | 1e-101 | -2.332e+02 | 3.65% | 0.26% | 29.0bp (63.6bp) | SpiB(ETS)/OCILY3-SPIB-ChIP-Seq(GSE56857)/Homer(0.792) More Information \| Similar Motifs Found | motif file (matrix) |
| 11 | A G T C A C G T A C G T A G T C A G T C A G C T A C G T | 1e-89 | -2.065e+02 | 22.88% | 11.99% | 51.5bp (99.1bp) | GCR2/MA0305.1/Jaspar(0.867) More Information \| Similar Motifs Found | motif file (matrix) |
| 12 | A C T G C T A G C T G A C T G A T G C A A C T G A C T G | 1e-88 | -2.045e+02 | 29.46% | 17.22% | 44.5bp (79.5bp) | PCBP1(KH)/Mus\_musculus-RNCMPT00239-PBM/HughesRNA(0.849) More Information \| Similar Motifs Found | motif file (matrix) |
| 13 | A G T C A G T C T G A C G A T C A C G T G A C T T A G C A T G C G T A C G A T C C G A T A G C T A G T C G T A C | 1e-82 | -1.908e+02 | 5.19% | 0.96% | 54.6bp (65.5bp) | RO3G\_00049(RRM)/Rhizopus\_oryzae-RNCMPT00205-PBM/HughesRNA(0.742) More Information \| Similar Motifs Found | motif file (matrix) |
| 14 | A C G T A T G C A C G T A C G T A G T C A G T C G C A T T A G C A C G T G C A T T C A G C G A T A C G T A G T C | 1e-79 | -1.832e+02 | 4.85% | 0.87% | 56.2bp (64.6bp) | SPIC/MA0687.1/Jaspar(0.745) More Information \| Similar Motifs Found | motif file (matrix) |
| 15 | A G T C A C G T A C G T T A G C G A C T A C G T T A G C C G A T A G T C G A C T A C G T A T G C A G T C A C G T | 1e-78 | -1.807e+02 | 4.85% | 0.89% | 48.4bp (76.2bp) | EWSR1-FLI1/MA0149.1/Jaspar(0.626) More Information \| Similar Motifs Found | motif file (matrix) |
| 16 | A G T C A C G T A T G C G T A C C G A T G A T C A G T C A C G T T G C A T A G C C G A T A T G C G T A C A C G T | 1e-65 | -1.514e+02 | 2.89% | 0.32% | 43.2bp (2.7bp) | SF2(RRM)/Drosophila\_melanogaster-RNCMPT00066-PBM/HughesRNA(0.635) More Information \| Similar Motifs Found | motif file (matrix) |
| 17 | A C T G C T A G C T G A C T A G C T G A C G T A C T A G C T A G C T G A | 1e-52 | -1.206e+02 | 11.57% | 5.54% | 52.5bp (92.6bp) | SRSF1(RRM)/Homo\_sapiens-RNCMPT00163-PBM/HughesRNA(0.755) More Information \| Similar Motifs Found | motif file (matrix) |
| 18 | G T A C A T C G A C T G C G T A A G T C A C G T C G A T A T G C A T G C | 1e-41 | -9.625e+01 | 2.59% | 0.48% | 56.4bp (77.5bp) | GCR1(MacIsaac)/Yeast(0.790) More Information \| Similar Motifs Found | motif file (matrix) |
| 19 | C T G A C T G A A G T C C T G A A C T G A T G C C G T A | 1e-41 | -9.470e+01 | 32.84% | 23.90% | 55.2bp (72.2bp) | ACE2/ACE2\_YPD/2-SWI5(Harbison)/Yeast(0.793) More Information \| Similar Motifs Found | motif file (matrix) |
| 20 | C T A G G A T C G T C A G C A T G A T C A G T C C G T A T C G A C T G A A G C T C G A T T A C G C T A G | 1e-40 | -9.398e+01 | 1.41% | 0.09% | 25.4bp (68.9bp) | HNRNPR(RRM)/Gallus\_gallus-RNCMPT00288-PBM/HughesRNA(0.686) More Information \| Similar Motifs Found | motif file (matrix) |
| 21 | A G T C A G C T T C G A A C T G G A T C A G T C C G T A T C A G C G T A A C T G G T A C C G T A | 1e-40 | -9.316e+01 | 1.39% | 0.09% | 51.8bp (63.4bp) | ZNF519(Zf)/HEK293-ZNF519.GFP-ChIP-Seq(GSE58341)/Homer(0.620) More Information \| Similar Motifs Found | motif file (matrix) |
| 22 | C T A G C G T A T C A G A G C T G T A C A G C T A C G T A G C T C G T A C T A G C T A G C A T G G A C T | 1e-38 | -8.847e+01 | 1.16% | 0.05% | 54.9bp (57.5bp) | AT4G12670(MYBrelated)/col-AT4G12670-DAP-Seq(GSE60143)/Homer(0.684) More Information \| Similar Motifs Found | motif file (matrix) |
| 23 | A C G T G A T C G A T C A C G T A G T C G T A C C G A T A G T C A C G T A C G T G T A C A G T C C G A T C A T G | 1e-33 | -7.767e+01 | 3.40% | 1.01% | 50.8bp (80.6bp) | ZNF189(Zf)/HEK293-ZNF189.GFP-ChIP-Seq(GSE58341)/Homer(0.725) More Information \| Similar Motifs Found | motif file (matrix) |
| 24 | A C G T A G C T T A G C G C T A G T C A G T A C G C T A A C G T C G T A T C A G C G A T T C G A A G T C | 1e-33 | -7.627e+01 | 1.34% | 0.13% | 69.2bp (63.2bp) | Tb\_0216(RRM)/Trypanosoma\_brucei-RNCMPT00216-PBM/HughesRNA(0.652) More Information \| Similar Motifs Found | motif file (matrix) |
| 25 | A T G C A G C T G A T C A C G T A C G T C A T G G T A C A G T C G A C T C T A G C G T A G A C T | 1e-32 | -7.447e+01 | 1.91% | 0.33% | 45.7bp (65.9bp) | SD0002.1\_at\_AC\_acceptor/Jaspar(0.712) More Information \| Similar Motifs Found | motif file (matrix) |
| 26 | A G C T A G C T G A C T G T C A A C G T T G A C C G T A C T A G C G T A A C G T T G A C C G A T C G T A | 1e-31 | -7.147e+01 | 1.08% | 0.07% | 75.6bp (50.9bp) | AGP1(GATA)/Nicotiana tabacum/AthaMap(0.688) More Information \| Similar Motifs Found | motif file (matrix) |
| 27 | G A C T G A C T T G C A G C A T C G T A A G T C G T A C G A C T C T G A C A T G C T G A T C G A T C G A | 1e-29 | -6.715e+01 | 2.06% | 0.45% | 48.2bp (68.7bp) | PB0006.1\_Bcl6b\_1/Jaspar(0.709) More Information \| Similar Motifs Found | motif file (matrix) |
| 28 | A G T C A C G T C G A T A G T C A C G T A C G T A G C T C G A T A C G T T A G C A C G T A C G T A G T C A C G T | 1e-29 | -6.704e+01 | 1.81% | 0.34% | 42.4bp (36.1bp) | TRA2(RRM)/Drosophila\_melanogaster-RNCMPT00078-PBM/HughesRNA(0.674) More Information \| Similar Motifs Found | motif file (matrix) |
| 29 | A G C T G A T C C G A T G A C T C G A T A T G C A G C T G A T C C G A T A C G T C T A G A G T C A G T C | 1e-27 | -6.317e+01 | 1.47% | 0.23% | 40.1bp (59.5bp) | SRSF10(RRM)/Homo\_sapiens-RNCMPT00019-PBM/HughesRNA(0.695) More Information \| Similar Motifs Found | motif file (matrix) |
| 30 | A G T C C G T A A G T C A C G T G C A T A C G T A G T C C T G A A G T C | 1e-27 | -6.279e+01 | 4.12% | 1.62% | 66.5bp (67.3bp) | PRDM1/MA0508.1/Jaspar(0.815) More Information \| Similar Motifs Found | motif file (matrix) |
| 31 | T A C G G T C A C T G A G T A C G C A T T A G C G C T A G A T C G C A T T G A C G C T A G T A C | 1e-25 | -5.879e+01 | 1.57% | 0.29% | 50.7bp (71.9bp) | z/dmmpmm(Pollard)/fly(0.695) More Information \| Similar Motifs Found | motif file (matrix) |
| 32 | C T A G C G T A A T C G A G T C C T G A G T C A A C G T A G T C C T A G T A C G | 1e-22 | -5.250e+01 | 0.86% | 0.07% | 46.6bp (53.2bp) | NFYB/MA0502.1/Jaspar(0.689) More Information \| Similar Motifs Found | motif file (matrix) |
| 33 | C T G A A C G T A G T C C G T A G A C T C T G A A C G T A G T C C T G A C G A T A G T C C G T A C T A G | 1e-22 | -5.250e+01 | 0.86% | 0.07% | 34.9bp (40.8bp) | RBM47(RRM)/Gallus\_gallus-RNCMPT00279-PBM/HughesRNA(0.649) More Information \| Similar Motifs Found | motif file (matrix) |
| 34 | G T A C C T G A A G T C C G A T T A G C G T C A G T A C G A C T T C G A G A C T A G T C | 1e-22 | -5.176e+01 | 1.22% | 0.19% | 48.4bp (71.0bp) | STZ(C2H2)/colamp-STZ-DAP-Seq(GSE60143)/Homer(0.701) More Information \| Similar Motifs Found | motif file (matrix) |
| 35 | G T A C G A T C C G A T A G C T A G C T G C A T C T G A G C A T C G A T G C A T G T A C G C A T | 1e-21 | -4.870e+01 | 1.51% | 0.33% | 44.6bp (61.4bp) | T1ISRE(IRF)/ThioMac-Ifnb-Expression/Homer(0.723) More Information \| Similar Motifs Found | motif file (matrix) |
| 36 | C G T A A G T C C G T A C G A T C T A G T C G A C A G T G A T C G A C T G C A T T G C A A C G T | 1e-20 | -4.664e+01 | 0.90% | 0.11% | 47.1bp (64.2bp) | Tv\_0259(RRM)/Trichomonas\_vaginalis-RNCMPT00259-PBM/HughesRNA(0.676) More Information \| Similar Motifs Found | motif file (matrix) |
| 37 | T C G A C G T A A T G C C G T A C G T A A G T C A G T C C G T A A T C G G T A C A C G T A G T C C G T A | 1e-19 | -4.465e+01 | 0.69% | 0.05% | 41.8bp (57.2bp) | YBX2(CSD)/Homo\_sapiens-RNCMPT00084-PBM/HughesRNA(0.653) More Information \| Similar Motifs Found | motif file (matrix) |
| 38 | A G T C G C T A A G C T C G A T C G A T C T A G T G A C | 1e-19 | -4.396e+01 | 38.46% | 32.06% | 57.5bp (68.5bp) | Sf3b4(RRM)/Danio\_rerio-RNCMPT00224-PBM/HughesRNA(0.785) More Information \| Similar Motifs Found | motif file (matrix) |
| 39 | C T G A A G C T C T A G C T G A A G C T C G A T A C T G C A G T C G T A C G T A A C T G C G A T | 1e-18 | -4.179e+01 | 0.96% | 0.15% | 70.6bp (72.9bp) | ATHB-12/MA0950.1/Jaspar(0.705) More Information \| Similar Motifs Found | motif file (matrix) |
| 40 | G C A T A G T C C T A G A C G T C G A T A G C T C T G A C A G T A T G C C T G A | 1e-17 | -4.123e+01 | 2.28% | 0.81% | 69.7bp (68.0bp) | KHDRBS3(KH)/Homo\_sapiens-RNCMPT00034-PBM/HughesRNA(0.761) More Information \| Similar Motifs Found | motif file (matrix) |
| 41 | G T A C G C A T A T C G C G A T A C G T A C T G A G T C A C G T A T C G A C G T C G A T A G T C A G C T A C G T | 1e-17 | -3.923e+01 | 1.04% | 0.20% | 47.8bp (69.3bp) | AR-halfsite(NR)/LNCaP-AR-ChIP-Seq(GSE27824)/Homer(0.704) More Information \| Similar Motifs Found | motif file (matrix) |
| 42 | C G T A C G A T G T A C T C A G A T C G T C A G G A T C G T C A C G T A | 1e-16 | -3.914e+01 | 1.10% | 0.22% | 47.0bp (66.3bp) | ARF2(ARF)/col-ARF2-DAP-Seq(GSE60143)/Homer(0.731) More Information \| Similar Motifs Found | motif file (matrix) |
| 43 | A G T C A C T G A C G T A C G T C G A T A G T C G T A C | 1e-16 | -3.896e+01 | 15.89% | 11.62% | 52.7bp (76.0bp) | STE12/MA0393.1/Jaspar(0.831) More Information \| Similar Motifs Found | motif file (matrix) |
| 44 | C T A G T G A C G T C A C T G A A C G T A C T G G C A T C T G A | 1e-16 | -3.754e+01 | 8.94% | 5.80% | 59.6bp (67.4bp) | RBM41(RRM)/Homo\_sapiens-RNCMPT00053-PBM/HughesRNA(0.814) More Information \| Similar Motifs Found | motif file (matrix) |
| 45 | C A T G G T A C C T G A C A T G G T A C T G C A T A C G G A T C G C T A T C G A | 1e-15 | -3.679e+01 | 4.67% | 2.50% | 53.4bp (71.6bp) | ACE2/ACE2\_YPD/2-SWI5(Harbison)/Yeast(0.688) More Information \| Similar Motifs Found | motif file (matrix) |
| 46 | A C G T A G T C G T C A A G C T C T A G C G T A A C T G G T C A C T G A A G T C | 1e-15 | -3.608e+01 | 1.57% | 0.47% | 50.6bp (71.7bp) | PB0194.1\_Zbtb12\_2/Jaspar(0.768) More Information \| Similar Motifs Found | motif file (matrix) |
| 47 | A T C G G C A T T C A G C T G A A G T C C G A T C A T G T C A G T C G A A G C T T G A C | 1e-15 | -3.586e+01 | 0.82% | 0.13% | 72.9bp (45.9bp) | RBM6(RRM)/Homo\_sapiens-RNCMPT00170-PBM/HughesRNA(0.718) More Information \| Similar Motifs Found | motif file (matrix) |
| 48 | A C G T A C G T C G T A A C G T A C G T A C G T A C T G C G T A C G T A A G T C C G T A A G T C | 1e-15 | -3.571e+01 | 0.43% | 0.02% | 60.3bp (31.5bp) | ct/MA0218.1/Jaspar(0.674) More Information \| Similar Motifs Found | motif file (matrix) |
| 49 | A T C G C G A T C G A T C T G A C G T A G C T A T G A C C G T A G A C T C G T A A T C G | 1e-14 | -3.265e+01 | 0.77% | 0.12% | 51.0bp (64.7bp) | AT3G25990(Trihelix)/colamp-AT3G25990-DAP-Seq(GSE60143)/Homer(0.758) More Information \| Similar Motifs Found | motif file (matrix) |
| 50 | C T G A C G A T A C G T G A C T C G T A C G T A A G T C C G T A | 1e-13 | -3.182e+01 | 10.35% | 7.22% | 61.4bp (65.4bp) | CG11617/dmmpmm(Noyes\_hd)/fly(0.926) More Information \| Similar Motifs Found | motif file (matrix) |
| 51 | A G C T G A C T C G A T T A C G A G T C A G C T C A G T C A G T T C A G G C A T A G C T A C G T C A T G G T C A | 1e-13 | -3.161e+01 | 1.34% | 0.40% | 52.2bp (69.3bp) | AT5G60130(ABI3VP1)/col-AT5G60130-DAP-Seq(GSE60143)/Homer(0.622) More Information \| Similar Motifs Found | motif file (matrix) |
| 52 | A C G T G C A T A C T G C G A T C G T A C G T A A T C G C G A T A C G T A G C T | 1e-13 | -3.144e+01 | 1.57% | 0.53% | 64.6bp (66.6bp) | PUM(PUF)/Drosophila\_melanogaster-RNCMPT00105-PBM/HughesRNA(0.708) More Information \| Similar Motifs Found | motif file (matrix) |
| 53 | C G T A C G T A A G T C C T A G G C T A A G T C T A G C C T G A C T A G T C G A | 1e-13 | -3.043e+01 | 0.77% | 0.14% | 39.4bp (63.3bp) | hkb/dmmpmm(Papatsenko)/fly(0.676) More Information \| Similar Motifs Found | motif file (matrix) |
| 54 | C G A T A C T G A G T C G T C A A C T G A C G T C G T A C G T A C G T A A C T G A C G T | 1e-13 | -3.024e+01 | 0.41% | 0.02% | 80.2bp (34.7bp) | Adof1(C2C2dof)/col-Adof1-DAP-Seq(GSE60143)/Homer(0.717) More Information \| Similar Motifs Found | motif file (matrix) |
| 55 | C G A T A G C T A G C T A G T C C G A T G T A C A G T C A C G T A G T C G T A C C G A T A G T C C G A T T C A G | 1e-12 | -2.808e+01 | 4.36% | 2.51% | 56.8bp (72.7bp) | eor-1/MA0543.1/Jaspar(0.736) More Information \| Similar Motifs Found | motif file (matrix) |
